# Insulin effect on glucose transport in thymocytes and splenocytes from rats with metabolic syndrome

**DOI:** 10.1186/1758-5996-2-64

**Published:** 2010-11-02

**Authors:** Roxana Carbó, Verónica Guarner

**Affiliations:** 1 Physiology Department, National Institute of Cardiology "Ignacio Chávez". Juan Badiano # 1, Col. Sección XVI, Tlalpan, C.P. 14080 México, D.F., México

**Keywords:** metabolic syndrome, insulin, lymphocytes, glucose transporters

## Abstract

Metabolic syndrome (MS) may comprise several clinical conditions such as obesity, diabetes and inflammatory disorders, which are characterized by metabolic imbalances. The study of glucose transport and regulation by insulin in lymphocytes is important, since the way they increase inflammation and susceptibility to infections are common in MS. We studied glucose internalization in isolated thymocytes and splenocytes, its regulation by insulin, and the role of three glucose transporters (Gluts) in control and in MS rats. Control glucose internalization and insulin responses were lower in splenocytes than in thymocytes. Control and insulin-induced glucose internalization in thymocytes declined with age, while transport by splenocyte continued to respond to insulin. Control thymocyte glucose internalization was blocked by antibodies against Glut 1 and 4, while the insulin response also was blocked by an anti-Glut 3 antibody. On four month old control and insulin-induced response, splenocyte transport was only blocked by Glut 1 and 4 antibodies. At six months splenocyte glucose internalization depended on Glut 1 and was less sensitive to the effects of an anti-Glut 4 antibody. In MS splenocytes the capacity of anti-Glut 1 antibodies to inhibit control and insulin-dependent glucose transport was less significant, and we found that in MS rats, glucose internalization was dependent on Glut 3 and Glut 4. In summary, the altered metabolic state present in MS rats shows signs of modulation of glucose internalization by the Glut1, Glut 3 and Glut 4 transporters, compared with its own age control.

## Background

Metabolic Syndrome (MS) should be considered as a cluster of mostly modifiable risk factors triggering a proinflammatory state, that provide a higher risk of the development of diabetes and cardiovascular diseases. Specific cardiovascular disease risk factors might include obesity, type 2 diabetes, hyperlipidemia, insulin resistance and hypertension. Some other alterations, such as a pro-coagulant state and pro-inflammatory signs, can be included [1]. The characteristic of the MS is that its clinical conditions share a metabolic imbalance, induce an excessive release of inflammatory mediators and have a marked stimulation of stress hormones and this, in turn, has profound effects on energy and substrate metabolism [2].

The immune system is crucial for the defense against organisms that cause infections and against toxic products; a single defect in any of its components can cause a breakdown in this defense system and lead to serious or fatal diseases. The consequences can be systemic infections, cancer, autoimmune disorders or metabolic impairments. In diabetic patients the incorrect management of sepsis, due to an inappropriate immune response, can cause excess inflammation, which can also decrease longevity [[3-5], and [6]].

Lymphocytes as part of the adaptive immune response are critical for normal immune functions [7]. These cells use glucose as a primary fuel source and a strict regulation of glucose is required to maintain immune homeostasis; they also divide rapidly and they have a high death rate. They further have the ability to respond to the presence of pathogens, shifting from a quiescent phenotype to a highly active state within hours of stimulation [[5,6], and [8]]. During activation, lymphocytes must dramatically alter their metabolism; they are able to increase their oxidative phosphorylation enough to supply their need and must therefore increase the rate of glycolysis. In this way many factors such as metabolic requirements, hormones and external signals modify their glucose consumption [8]. The study of glucose transport in lymphocytes and its regulation by insulin is important in the MS, since the exacerbated immune response participates in physiological and pathological conditions of diabetes mellitus type II (T2DM), which is an important component of this syndrome [9]. The reason for the increased susceptibility of diabetic patients to persistent infections is not fully understood, but it is known that sepsis or infection, as in diabetic ulcers, raises cytokine secretion, which exacerbates damage, produces insulin resistance and diminishes lymphocyte proliferation. Sepsis injury and lymphocyte response to it can come from alterations in the intracellular calcium homeostasis [10]. Some of the altered functions of diabetic lymphocytes can be restored by administration of insulin [11] and a beneficial effect from this hormone again depends on the calcium homeostasis. Other changes such as reduced production of interleukins 2, 6 and 10 (IL-2, IL-6, and IL-10) are induced by raised concentrations of glucose [12].

Glucose transport into the cell, its regulation and the mechanism of action of the hormones involved, varies among different tissues and organs as well as during development [13,14]. Glucose transport also varies according to the rate of division and to metabolic necessities. Therefore differences in glucose transport between immature and mature lymphocytes might be expected.

Glucose is transferred across the plasma membrane by facilitated diffusion along a concentration gradient involving transport proteins called glucose transporters (Gluts), which have been characterized according to their transport properties and to the requirements of the tissue in which they are expressed [15]. There are 14 isoforms, which exhibit different specificities, kinetic properties and tissue expression profiles. In lymphocytes, expression of Glut 1, Glut 4 and Glut 3 molecules has been detected [16-18]. To prevent death, lymphocytes over-express glucose transporters, especially Glut 1, which is the major glucose transporter in this type of cell [19,20].

Regulatory factors, such as insulin level and hypoxia [21-23], enhance the expression of Glut1 transporters and move Glut 4 transporter molecules from small tubulo-vesicular elements near the endoplasmic reticulum into the plasma membrane [24,25]. Also the calcium-calmodulin complex may be a part of the intracellular signaling pathway of this regulatory factor.

In this study, we used a rat model of MS developed in our institution by Baños *et al*. [26]. The characteristics of these rats are: moderate elevation of blood pressure, hypertriglyceridemia, hyperinsulinemia, excessive visceral adipose tissue, renal damage, high vascular reactivity, high inflammatory state, but not hyperglycemia [27-30].

Due to the pro-inflammatory state and metabolic disorders present in the MS, the aim of this study was to study the glucose transport at two different stages of maturation of the lymphocytes in these animals and the participation of three known glucose transporters in these types of cells. Our interest increased due to the inflammatory state and the non-diabetic condition of this model. We studied the modulation of glucose transference in thymocytes and splenocytes mediated by three different glucose transporters (Glut 1, 3 and 4), using antibodies to block glucose internalization, to possibly define the participation of the glucose transporters in the MS.

## Methods

### Animals

#### Control animals

Two control groups of male Wistar rats (*Rattus norvegicus*) were studied; young 4 month old rats (150-200 g) and medium age six month old rats (400-500 g). Control animals: 4 month old animals were used to measure the thymus activity, which declines with age [31], and 6 months old, because the MS model is fully achieved in rats at six months of age. They were fed after weaning, with normal rodent diet and water *ad libitum*. At the end of treatment period, the rats were killed by decapitation to obtain the blood, spleens and thymuses for experiments. Decapitation was chosen to avoid the interference by any anesthetic and to allow blood draining. Serum was obtained by blood centrifugation.

#### Metabolic Syndrome animals (MS)

Male Wistar rats were fed from weaning (50-80 g) up to six months of age, with normal rodent diet and a solution of 30% sucrose as the only liquid source. At the end of the treatment these animals were medium age adults (400-500 g), which were killed by the same procedure as the other groups. To determine the MS, their weight, blood glucose and triglyceride concentration, blood pressure and insulin concentration were measured. These variables were also determined to the control animals.

#### Blood pressure measurements

Systolic arterial blood pressure was measured in conscious animals using the tail-cuff method; the cuff was connected to a pneumatic pulse transducer (Narco bio-systems Inc., Healthdyne Co.) and a programmed electro sphygmomanometer. The mean was calculated. The recordings were taken of six independent determinations by means of a Grass polygraph (model 79, Grass Medical Instruments, Quincy, MA).

#### Blood parameter determination

After overnight fasting (12 hours), the animals were killed by decapitation and blood was collected. It was spun and the serum was separated by centrifugation at 15,000 rpm during 15 min at room temperature and stored at -70°C until needed.

##### Serum insulin

Serum insulin was determined using a commercial radioimmunoassay (RIA) kit specific for rat (Linco Research, Inc. Missouri, USA); the sensitivity was of 0.1 ng/mL and intra- and inter-assay coefficients of variation were 5 and 10%, respectively.

##### Serum glucose

Glucose concentration was assayed using an enzymatic Kit SERA-PAK^R ^Plus (Bayer Corporation, Sées, France).

##### Serum triglycerides

Triglycerides (TGs) were determined by commercially available procedures (Randox, Laboratories LTD, Antrim, United Kingdom).

##### Serum cholesterol

Cholesterol was determined by commercial enzymatic procedure SPINREACT cholesterol -LQ (Spinreact S. A. Gerona Spain).

##### Homeostasis model assessment (HOMA)

HOMA was used as an index to measure the degree of insulin resistance and was calculated by the formula: (insulin (μU/ml) × glucose (in mmol/L)/22.5) [32-34].

### Cell Preparation

Immediately after blood draining, spleens and thymuses were dissected and placed in cold Tyrode solution. All solutions were bubbled with 95%O_2_-5%CO_2_, pO_2 _160 and pCO_2 _21.3 mmHg. The organs were extracted from the control and MS rats. Spleens and thymuses were cut separately into small pieces (2 mm^2^) and filtered through fine gauze and then centrifuged 3 min at 1000 rpm. In the spleen, erythrocytes were lysed by adding 3 ml of a lysis solution to the pellet; (1 part of Tris (0.17 M) and 9 parts of NH_4_Cl (0.16 M)). The cells were gently agitated in this buffer for 1 min and centrifuged again. They were washed twice with Tyrode to remove the lysis buffer. Thymus cells were filtered and washed once and resuspended in Tyrode. Macrophages from both cell lines were removed by incubating the cell at 37°C for 30 min in plastic Petri dishes. The cells were then washed twice in fresh Tyrode solution by centrifuging 3 min at 1000 rpm and resuspending the pellet. Cellular viability was determined by the percentage of cells that excluded 0.3% Trypan Blue (Gibco-BRL) [35] in Tyrode in an improved Neubauer haemocytometer 1/10 mm deep (Clay-Adams, Parsippany, NJ, USA). The viability was of 76.5 ± 1.2%. There were no significant variations of cell viability after the incubation period, so the changes observed were due to the treatment and not to the variability in the cell population.

### Glucose Transporter Protocol

Cells were centrifuged again and aliquots of 5 × 10^6 ^cells (approximately 100 μl of the pellet) were placed in Eppendorff tubes, centrifuged and the supernatant substituted by 100 μl of one of the following different solutions added to each tube: **1) **Tyrode solution (Tyr), **2) **Tyrode solution with 50 mU/L insulin (Insulin) (Eli Lilly), approximately the postprandial dose reached in plasma, **3) **Tyrode solution with 1 μM trifluoperazine (TPZ) (ICN Biochemicals, Aurora OH, USA), a calcium-calmodulin complex blocker [36,37]; **4) **Tyrode containing commercial anti-Glut1 antibodies (G1), directed against the extracellular domain (1:1000), (Santa Cruz, Biotech), raised in goat, **5) **Tyrode containing commercial anti-Glut 3 antibodies (G3), directed against the extracellular domain (1:10,000), (Santa Cruz, Biotech), when used in thymocytes and (1:5,000) when used in splenocytes, **6) **Tyrode containing commercial anti-Glut 4 antibodies (G4), directed against the extracellular domain (1:10,000), (Santa Cruz, Biotech), raised in goat, **7) **Tyrode solution plus an Affinipure goat anti-rabbit serum(serum), at the same concentration as the antibodies used to block the glucose transporters (negative control) (Jackson ImmunoResearch Laboratories, West Grove, PA, USA), **8) **Tyrode solution with 0.1 μM cytochalasin B (Cyto) (Sigma, St. Louis MO), a glucose transporter blocker (positive control) [38]. All doses were chosen after obtaining a dose-response curve in young and healthy animals. The tubes were incubated for 1 h at 37°C with gentle agitation. The cell suspensions were constantly shaken, and at the end the suspensions were agitated again, so that glucose concentration was homogeneous. After the incubation period the cells were centrifuged at 1000 rpm for 3 min. Glucose concentration in the initial solution and in samples of the supernatant after 60 min of incubation was measured by the glucose oxidase method.

### Glucose Supernatant Determination

After the incubation time, 2 μl of the supernatants were added to 200 μl of a glucose assay, (Diagnostic Chemicals Limited, Oxford Co) to measure the glucose concentration by the glucose oxidase reaction [39], in a Benchmark Plus microplate spectrophotometer (Bio-rad, Hercules, CA) at 505 nm.

### Ethics

All animals were lawfully acquired and the manipulation of all animals in the research was carried out in accordance with "The European Commmission Enviroment" (Declaration of Helsinki): EC Directive 86/609/EEC for animal experiments [40]. It also received the approval from the Ethics Committee of the National Institute of Cardiology. All reagents used were of the highest grade available.

### Statistical Analysis

Data were reported as micrograms of glucose subtracted from the incubation media per gram of fresh tissue per hour. Means and standard errors of at least 20 experiments were calculated. ANOVA was used to evaluate the differences on glucose internalization produced by the cell populations. The Mann Whitney test was used to determine which differences were statistically significant considering p < 0.05 as significant. Student's t test was used to compare control and insulin responses.

## Results

### Metabolic Syndrome Model

After six months of drinking a high sucrose solution the animals develop many of the characteristics of the MS, showing similar symptoms to the fructose-fed model developed by Reaven [41]. The animals have moderately elevated blood pressure, hypertriglyceridemia, hyperinsulinemia, central adiposity, insulin resistance, but not hyperglycemia. Eventually, some of these animals showed altered lymphocyte functionality manifested as respiratory infections or abscesses. When this happened the animals were discarded. We only used healthy specimens. MS rats also have smaller surviving rates. Table 1 shows variables in these animals.

**Table 1:** Physiological variables measured on control and MS rats

	4 MONTHS CONTROL	6 MONTHS CONTROL	MS
Body weight (g)	250 ± 10.0	521.0 ± 6.2	530.0 ± 12.9
Intra-abdominal fat (g)	3.2 ± 0.8	4.3 ± 0.8	14.8 ± 4.0*
Blood Pressure (mmHg)	104 ± 2.0	110 ± 1.1	148 ± 2.9*
Total Cholesterol (mg/dl)	70.0 ± 1.6	74.7 ± 1.7	72.9 ± 2.4
Triglycerides (mg/dL)	51.6 ± 4.2	51.0 ± 5.8	109.6 ± 12*
Glucose (mmol/L)	5.76 ± 0.4	5.9 ± 0.3	5.38 ± 0.4
Insulin (μU/ml)	9.0 ± 2.8	6.5 ± 0.9	24.2 ± 5.7*
HOMA	1.5 ± 0.8	1.7 ± 0.5	5.2 ± 2.1*

Values are mean ± s.e.m. n = 20; *p < 0.01

### Insulin Effect on Glucose Internalization of Control and MS Rat Lymphocytes

#### Thymocytes

**Figure 1:**
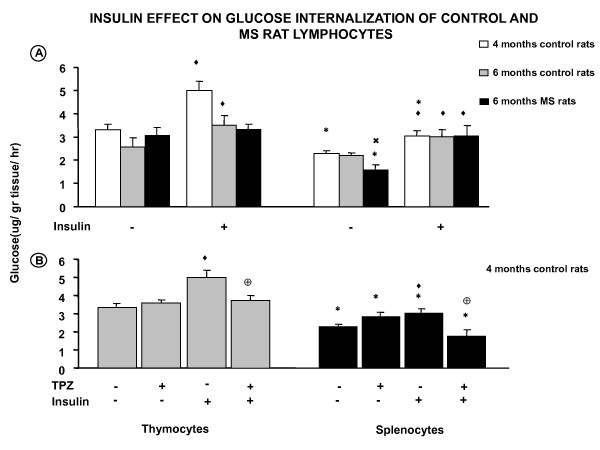
**Insulin effect on glucose internalization of control and MS rat lymphocytes**. **A**) Comparative glucose transport in control and MS thymocytes and splenocytes, with and without insulin. **B**) Experiments with TPZ as a control of the possible pathway of the insulin effect. * *p *< 0.05 comparing the two types of cells; ^♦ ^*p *< 0.05 insulin effect when compared to control values incubated with Tyrode; ^♥ ^*p *< 0.05 comparing between ages; **^× ^***p *< 0.05 MS effect of the MS compared to its 6 months control; ⊕ *p *< 0.05 compared with its insulin control.

Glucose internalization was significantly larger in immature thymocytes and they incorporated 3.31 ± 0.23 glucose μg/fresh tissue g/h. There were differences between 4 and 6 months of age in control thymocytes, the 4 months old cells being higher glucose consumers. At the stage of 4 months the value of glucose transport in thymocytes was 51%; meanwhile at the age of 6 months thymocytes decreased their glucose transport by 15% (Figure 1A).

Insulin significantly increased glucose transport in thymocytes at the age of 4 and 6 months old, but glucose transport diminished with time; the insulin-induced response at 6 months old was reduced to 66.4%.

Basal glucose internalization in thymocytes from MS rats was the same as in 4 and 6 month old controls, but they stopped responding to insulin (Figure 1A).

#### Splenocytes

In cells isolated from the spleen the glucose internalization was not as high as in thymocytes. However this insulin effect was more pronounced (19%) in immature thymocytes than in splenocytes; these differences decreased at the age of 6 months. Splenocytes incorporated 2.29 ± 0.12 glucose μg/fresh tissue g/h. and did not change their transport through time. The insulin effect in these cells was present at both ages (Figure 1A).

MS splenocytes decreased their glucose transport, but they had a greater insulin-induced effect compared with its own age control (Figure 1A).

Insulin effect on both cell populations was reduced by a dose of 1 μM of trifluoperazine, which is a calcium-calmodulin inhibitor. Therefore calcium-calmodulin could be one of the possible pathways of the insulin effect. Trifluoperazine had no effect on control glucose transport (Figure 1B).

### Glucose Transporter Participation in Basal and Insulin-induced Glucose Internalization of Young Rat Lymphocytes

#### Thymocytes

**Figure 2:**
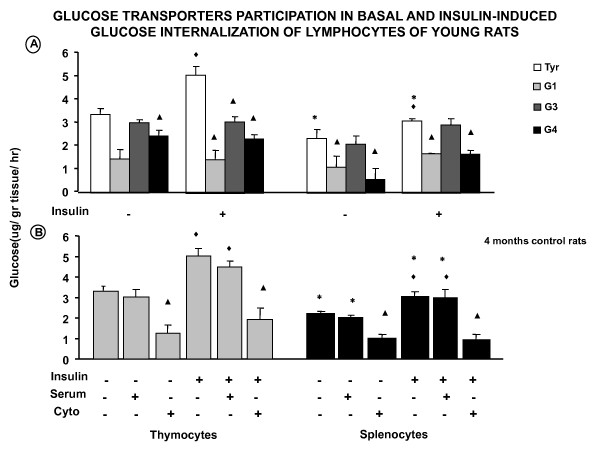
**Glucose transporter participation in basal and insulin-induced glucose internalization of young rat lymphocytes**. **A**) Effect of the three different antibodies against the glucose transporter on glucose transport of young (4 months) control cells and the insulin effect. **B**) Experiments in the presence of an unspecific serum without antibodies against the glucose transporters and in the presence of cytochalasin B (Cyto B), a non-selective glucose transporter blocker, * *p *< 0.05 comparing the two type of cells; ^♦ ^*p *< 0.05 insulin effect when compared to control values incubated with Tyrode; **▲ ***p *< 0.05 compared to its own Tyrode control.

At the age of 4 months, the participation of glucose transport molecules in both basal and insulin-induced glucose internalization was studied by incubating thymocytes (immature cells) in the presence of commercial anti-Glut1, anti-Glut 3 and anti-Glut4 antibodies. Antibodies against the Glut1 and Glut4 molecules significantly decreased basal and insulin-induced response. Anti-Glut 3 antibodies had no effect on the basal transport (Figure 2A). The higher insulin response of thymocytes was blocked by antibodies against the Glut 1 and Glut 4 transporters and also by antibodies against Glut 3 (Figure 2A).

Basal and insulin-induced glucose transport were significantly decreased when thymocytes were incubated with cytochalasin B. Control goat serum without anti-glucose transporter antibodies did not alter either basal or insulin-induced glucose internalization (Figure 2B).

#### Splenocytes

Glucose transporter molecule participation in both basal and insulin-induced glucose internalization was also studied in splenocytes (mature cells) in the same way as in thymocytes and at the same age. Control and insulin-induced glucose consumption by splenocytes was blocked by anti-Glut 1 and anti-Glut 4 but not by anti-Glut 3 antibodies (Figure 2A).

Basal and insulin-induced glucose internalization were significantly decreased when splenocytes were incubated with cytochalasin B. Control goat serum without anti-glucose transporter antibodies did not alter basal or insulin-induced glucose capture (Figure 2B).

### Splenocyte Control and Insulin-induced Glucose Internalization at Two Ages

Due to the fact that the stock of thymocytes is built up in early life and the thymus functions decline as age advances, we decided to continue to study splenocytes only. This decision was also made because the glucose transport at 6 months old and in the MS thymocytes declined and there was no response to insulin.

**Figure 3:**
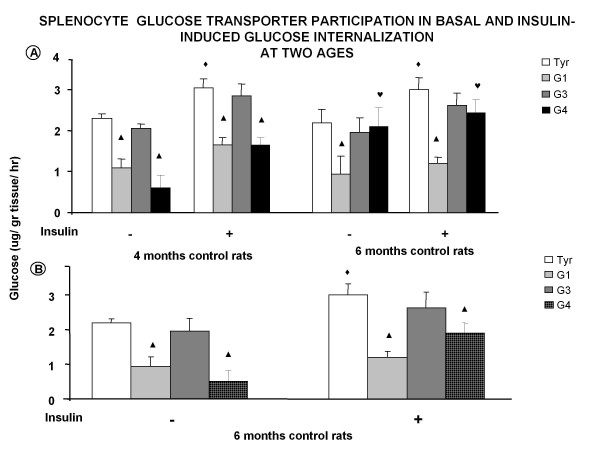
**Splenocyte glucose transporter participation in basal and insulin-induced glucose internalization at two ages**. **A**) Changes on the effect of the three different antibodies, against the glucose transporter, on glucose transport at 4 and 6 months with and without insulin. **B**) Given the different response to the anti Glut 4 antibodies there was the need to use a different antibody concentration (1:500); ^◆ ^*p *< 0.05 insulin effect when compared to control values incubated with Tyrode; **▲ ***p *< 0.05 compared to its own control; ^♥ ^*p *< 0.05 comparison between ages.

Different glucose transporter participation was observed at the two ages in splenocytes control cells. Splenocytes glucose internalization did not change through time, but the way in which cells captured glucose was different. At the age of 4 months, these cells incorporated glucose through Glut 1 and Glut 4 and insulin response promoted the entrance by the same transporters. At 6 months, Glut 1 also facilitated the entrance of glucose, but when the cells were incubated with the antibody against Glut 4 (1:10,000) it no longer participated, although a tendency to do so by insulin was observed. In the event, we concluded that the transporters could be modified by age (Figure 3A).

Then the cells were incubated with a higher concentration of anti Glut 4 antibodies (1:500) and a blockade in both cases was observed (control and insulin). The behavior of Glut 3 did not change with age (Figure 3B).

### Splenocyte Glucose Transporter Participation in Glucose Internalization with and without Insulin on the MS Model

**Figure 4:**
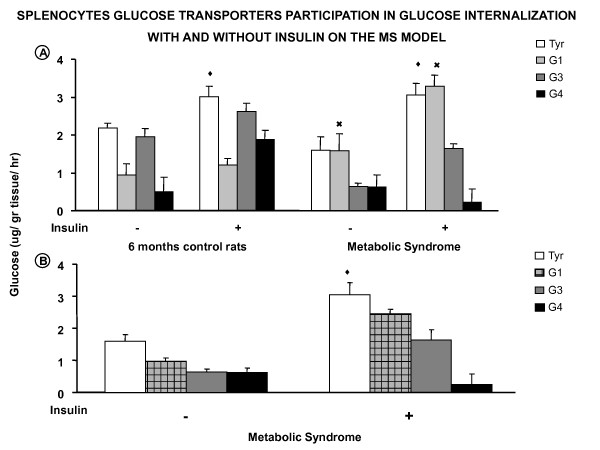
**Splenocyte glucose transporter participation in glucose internalization with and without insulin on the MS model**. **A) **Changes on the effect of the three different glucose transporter antibodies, on control and MS glucose transport are shown. Anti-Glut 3 antibodies responded in the MS splenocytes only. **B) **Given the different response to the anti Glut 1 antibodies there was also the need to use a different antibody concentration (1:500). Anti Glut 4 concentration used in the MS was the same (1:500) as in the previous experiments in control of six months old animals. ^◆ ^*p *< 0.05 insulin effect when compared to control values incubated with Tyrode; **▲ ***p *< 0.05 compared with its own control; **^× ^***p *< 0.05 MS effect compared with its 6 months control.

Glucose transport in MS animals is lower compared with control animals of the same age, but the response to insulin is enhanced. In this model, it seems that Glut 1 does not mediate basal glucose intake, but when an increase in the antibody concentration was used (1:500), a blockade was observed, as well as in the insulin response. This might suggest that in this condition the splenocytes need more glucose transporter in the membrane. Under control conditions, the participation of Glut 4 in the MS cells is blocked, with the same concentration (1:500) of antibodies as previously used in the 6 month old animals. When the MS splenocytes were stimulated by insulin at the same concentration of antibodies against Glut 4, the blockade was complete. This suggests that the quantity of antibody was sufficient to block the response. An activation of Glut 3 in the basal and insulin-induced transport was now observed in the MS model (Figure 4).

## Discussion

Lymphocytes, as part of the adaptive immune system, are critical for normal immune functions [7]. They require glucose as a primary fuel source and strict regulation of glucose is required to maintain immune homeostasis. Impairment of glucose transport in splenocytes and thymocytes and its regulation by insulin is a common feature of human diabetes, enhancing susceptibility to infections [9]. Although the reason for increased susceptibility of diabetic patients to persistent infections is not fully understood, the administration of insulin restores some of the altered functions of diabetic lymphocytes [11]. Raised concentrations of glucose also induce changes such as reduced production of interleukins [12]. In this paper we studied and compared glucose transport and insulin effect in immature thymocytes and mature splenocytes of control animals and MS animals.

In accordance with the observation that glucose transport changes during development and maturation in many cell types [13,14], we found that thymocytes show a very important difference in their effect on glucose transport when compared with splenocytes. This can be explained by the fact that thymocytes are immature cells that might be in the process of developing and therefore the metabolic demand increases, due to the fact that these cells initiate their activation, cell growth program and proliferation [42]. T cells exit the thymus and enter the peripheral circulation as small quiescent cells (resting state), they consume glucose at a low rate and consumption is limited by the availability of trophic signals rather than by nutrient availability [8]. These cells, due to their ability to respond rapidly in the presence of pathogens or inflammatory events, shift from a quiescent phenotype to a highly active state within hours of stimulation. This result is similar to the changes in glucose consumption in other blood cells such as erythrocytes [14].

Glucose enters cells by facilitated diffusion [43] via glucose transporter molecules [15].

Lymphocytes express Glut 1, Glut 3 and Glut 4 molecules [16-18]. In this paper we observed that both basal and insulin-induced glucose transport were inhibited by cytochalasin B, a molecule that binds covalently and in a reversible manner to all glucose transporters [38]. Anti-Glut1 and anti-Glut4 antibodies blocked the insulin-induced response, except in thymocytes, where Glut 3 participated in the insulin-induced response. These results suggest the participation of other transporter mediating insulin-induced glucose transport in thymocytes. Glut proteins are highly regulated in physiological as well as pathophysiological states. Levels of regulation include gene transcription, protein expression and degradation, cellular distribution, translocation and fusion and also Glut activity [44].

Regulation of glucose transport is also modified during development [13,14]. The capacity to respond to regulatory factors such as insulin has been studied in lymphocytes [8,45-47], but there are very few reports on immature thymocytes [48]. In this paper we show that incubation in the presence of insulin resulted in a significant increase in glucose capture both in splenocytes and thymocytes.

Basal and insulin-induced glucose internalization by thymocytes declines after some time and this can be due to thymus atrophy. The stock of T lymphocytes is built up in early life, so the function of the thymus is diminished in adults. In elderly adults it consists of fatty tissue. However, the thymus still functions as an endocrine gland that stimulates the immune system and this phenomenon is related to post-receptor defects in the mechanism of insulin-mediated glucose uptake in target tissues [31]. In the case of MS thymocytes there is a tendency to reduced glucose transport and no response to insulin. Age and obesity might explain the phenomenon, as well as the glucose imbalance. Armoni et al [49] found that Glut 4 stops being expressed in adipocytes with aging and this can also happen in thymocytes. Armoni also mentions that obesity is a factor that diminishes the expression of Glut 4 in adipocytes and our MS animals have a clear abdominal obesity.

Splenocytes formed in the bone marrow are naive and have never been exposed to antigens; they get activated and migrate to lymphoid organs. This route is a very high glucose-consuming process. In splenocytes a movement of the glucose transporter 4 with aging is observed and this can be explained by the fact that this transporter has a developmental expression. It has been reported that this Glut is regulated, in a tissue-specific manner, by a developmental program at early stages in different tissues and this program may coordinate the expression of other proteins of metabolic importance; therefore, it should not be surprising that this change in expression might also happen at later stages of development to adjust to metabolic changes [50]. B cells also express more Glut 4 with age and this might be due to the fact that cells get more hypoxic as they get older. Hypoxia has been reported as a Glut 4 expression promoter [21, 22, 51 and 52].

In the present paper we studied the participation of the calcium-calmodulin complex in insulin increases in glucose internalization through its inhibition, by using trifluoperazine [36,53]. It has been reported that subsequent to the recognition of insulin at the cell surface, intracellularly located glucose transporters are transported to the cell membrane, whereupon they increase sugar uptake [54]. For these events to occur, the hormone-receptor complex must release the signals that induce incorporation of transporters into the plasma membrane. Since transporter incorporation to the plasma membrane is due to a vesicle fusion mechanism similar to the exocytosis processes, calcium [54,55] and calmodulin [37] have been proposed as doorways and regulatory agents. On the other hand, glucose and calmodulin have also been found to regulate calcium uptake in insulinoma glucose-responsive cells [56]. Therefore, it would be possible that trifluoperazine could block the increase in glucose transport. When lymphocytes were incubated simultaneously with insulin and trifluoperazine, the increase in glucose internalization induced by insulin was decreased. These results indirectly support the role of Ca^2+ ^and calmodulin as the intracellular messenger of the insulin receptor complex, since calmodulin must interact with Ca^2+ ^to induce vesicle fusion to the plasma membrane. It has also been previously reported that an increase in intracellular calcium level leads to increases in cellular proliferation and secretion of intracellular products [55].

MS is associated with abdominal obesity, blood lipid disorders, inflammation, insulin resistance and an increased risk of cardiovascular diseases. Our model concedes with many of the MS characteristics and the obesity that these animals develop may be a risk factor for deteriorating cellular immune function [57].

The fact that the MS cells decrease their glucose transport compared to control cells might be due to the phenomenon called immunosenescence, which covers numerous immune system changes with age [58]. The MS accelerates aging. In the MS model an activation of Glut 3 transporter in basal transport is present. The insulin-stimulated response in the MS animals persists and a movement of the glucose transporter 1 and an activation of the glucose transport 3 by the effect of insulin can also be observed. These rats have hyperinsulinemia and it has been reported that in muscle cells chronic insulin stimulation can activate the over expression of Glut 1 and Glut 3 by two different pathways [59]. Szablewski described that Glut 1 in diabetic patients is over expressed in lymphocytes [60]. Although our animals are not diabetic they have a metabolic disturbance.

We postulate that, in our model, there are glucose transporter movements due to the metabolic disorders that these animals suffer [26-29]. Fu et al [16] proposed that high Glut 1 and 3 expressions could provide cellular fuel for the immune response. They also proposed that high affinity to Glut 3 in other immune cells might allow cells to compete with pathogens for hexoses. The participation of glucose transporters 1 and 3 suggests that splenocytes have an increased metabolic need for glucose because they have a sustained immune response due to the MS state or, possibly, because another cell population is activated. The possible decrement in the expression of Glut 4 after insulin stimulation, in this model, can also coincide with the fact that Glut 4 decrease, with age and the metabolic process that these rats are going through, can be compared with an accelerated aging process. Besides, Cartee [61] found that Glut 4 protein decreases with age in cardiomyocytes, so this could be also true for the splenocytes.

## Conclusions

We conclude that in rat, thymocytes consume more glucose than splenocytes and respond better to regulatory factors such as insulin when they are at an active stage. Lymphocytes show three different glucose transporters: Glut1, Glut 3 and Glut4. Thymocytes use Glut 1 and Glut 4 for basal glucose consumption and the three transporters participate in the insulin-induced response. Meanwhile, in splenocytes, glucose transport is made by Glut 1 and Glut 4 in basal and in the MS the glucose transport is diminished and the three glucose transporters are activated. There is a regulation of the transporters, where Glut 1 raises its participation, Glut 3 is activated and Glut 4 diminishes its response in the insulin stimulation. The participation of the calcium-calmodulin complex, as a possible intracellular signaling pathway for insulin, could suggest part of the regulation of this hormone in the incorporation of glucose into the cell and in the glycemic control of these animals. These results could contribute to a better understanding of why in the MS the immune system is constantly activated to respond to persistent infections, through the regulation of glucose transport.

## Competing Interest Statement

The authors declare that they have no competing interests.

## Authors' contributions

VG suggested the idea and supervised the work; RC structured and performed the experiments and analyses of the data. Both authors participated in the elaboration of the manuscript and have read the complete manuscript and take responsibility for its content and completeness and understand that if the paper or part of the paper is found to be faulty or fraudulent they share responsibility.
